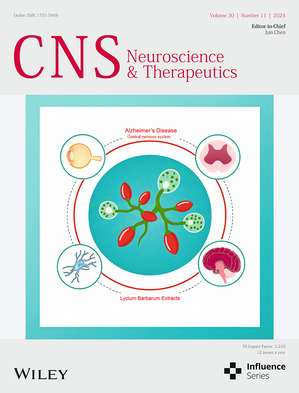# Front Cover

**DOI:** 10.1111/cns.70155

**Published:** 2024-12-04

## Abstract

Cover image: The cover image is based on the article *Lycium barbarum Extract Enhanced Neuroplasticity and Functional Recovery in 5xFAD Mice via Modulating Microglial Status of the Central Nervous System* by Zhongqing Sun et al., https://doi.org/10.1111/cns.70123.